# The efficacy and safety of non-resistance manual therapy in inpatients with acute neck pain caused by traffic accidents: A randomized controlled trial

**DOI:** 10.1097/MD.0000000000029151

**Published:** 2022-06-03

**Authors:** Suna Kim, Da-Hyun Kyeong, Min-Kyung Kim, Chang-Yeon Kim, Yoon Jae Lee, Jinho Lee, In-Hyuk Ha, Kyoung Sun Park

**Affiliations:** a Daejeon Jaseng Hospital of Korean Medicine, Seo-gu, Daejeon, Republic of Korea; b Jaseng Spine and Joint Research Institute, Jaseng Medical Foundation, Gangnam-gu, Seoul, Republic of Korea.; c Jaseng Hospital of Korean Medicine, Gangnam-gu, Seoul, Republic of Korea.

**Keywords:** acute neck pain, manual therapy, nonresistance manual therapy, protocol, randomized controlled trial, whiplash associated disorder

## Abstract

**Background:**

Neck pain and functional impairment are common complications of traffic accidents (TAs); however, the effects of manual therapy on these symptoms have rarely been studied in the literature. Thus, this randomized controlled trial aims to assess the effectiveness and safety of non-resistance manual therapy (NRT)—a treatment combining mobilization and pressure release techniques—on acute neck pain caused by TA.

**Method:**

This study will use a two-armed, parallel, assessor-blinded randomized controlled trial design and will be conducted in the Daejeon Jaseng Hospital of Korean Medicine in South Korea. One hundred twenty patients will be recruited and randomized into an integrative Korean medicine treatment (IMKT) + NRT group and IMKT group in a 1:1 ratio. The primary outcome is a change in the numeric rating scale for neck pain immediately after treatment on hospital day 5 compared to those at baseline. The secondary outcomes are numeric rating scale for radiating arm pain, visual analogue scale for neck pain and radiating arm pain, cervical active range of motion, neck disability index, Patient Global Impression of Change, Short Form-12 Health Survey, and Posttraumatic Stress Disorder Checklist for DSM-5.

**Discussion:**

The findings of this study on the effectiveness and safety of NRT will be helpful for patients with TA-induced neck pain in clinical practice and will provide evidence for developing relevant healthcare-related policies.

**Trial registration:**

This protocol has been registered at Clinicaltrials.gov (NCT04660175).

## 1 Introduction

Traffic accidents (TAs) are a common cause of neck pain, and cervical injury and relevant heterogeneous disorders caused by acceleration and deceleration of vehicles are collectively referred to as the whiplash syndrome.^[1]^ The presence of neck pain episodes is the best predictor of the onset of neck pain in the future, and a study that comprehensively analyzed 47 studies reported that approximately 50% of individuals with TA-related neck pain episodes suffered from neck pain even 1 year after the accident.^[2,3]^ Chronic neck pain can increase medical costs and incur social and financial losses due to disabilities. Some clinical guidelines recommend active exercise, passive joint mobilization, heat, ice and massage, electrotherapy, simple analgesics, and nonsteroidal antiinflammatory drugs for treating acute (≥12 weeks of onset) whiplash associated disorders.^[4]^

In Korea, many patients suffering from whiplash associated disorder seek Korean medicine treatment, although it is not covered by auto insurances. According to auto insurance medical cost statistics, Korean traditional medicine accounts for 48% of all medical costs.^[5]^ In Korean traditional medicine, neck pain is generally treated by acupuncture, electroacupuncture, herbal medicine, Chuna manual therapy, pharmacopuncture, bee-venom pharmacopuncture, cupping therapy, and Doin therapy.^[6]^

Chuna manual therapy is an advanced form of traditional Korean manual therapy that incorporates various forms of manual therapies used in other countries.^[7]^ Cervical nonresistance manual therapy (NRT) is a type of Chuna therapy comprising joint mobilization and pressure release techniques. Mobilization techniques are used in osteopathy and chiropractic therapies. Manual therapy using a pressure release technique is also performed in various countries worldwide.^[8–11]^

Many studies have suggested that compared to treatments for acute whiplash injury that involve joint fixation, treatments that do not involve joint fixation, such as movement within range of motion (ROM) and mobilization, are more effective in the long term.^[4,12–14]^ However, well-designed randomized controlled trials (RCTs) on these manual therapies are lacking. This parallel-group RCT aims to assess the effectiveness and safety of NRT on TA-induced neck pain. The hypothesis is that additional NRT with integrative Korean medicine treatment (IKMT) is superior to IKMT alone.

## 2 Method

### 2.1 Study setting

This is a protocol for a two-arm parallel RCT that will be conducted in the Daejeon Jaseng Hospital of Korean Medicine. This protocol was approved by the Institutional Review Board at the Daejeon Jaseng Hospital of Korean Medicine prior to patient recruitment (JASENG 2020-10-007). Further, the protocol has been registered at Clinicaltrials.gov (NCT04660175), and the progress of the study will continue to be updated. One hundred twenty inpatients of Daejeon Jaseng Hospital of Korean Medicine with TA-induced acute neck pain who will meet the inclusion criteria and sign an informed consent form will be enrolled. The patients will be recruited within the hospital. Once enrolled, the patients will complete seven visits from hospital day 1 and complete six surveys during their hospital stay and one survey after discharge.

### 2.2 Participants

#### 2.2.1 Inclusion criteria

1. Age 19 to 70 years
2. TA-induced acute neck pain that developed in the last 7 days
3. Hospitalization due to acute neck pain
4. Numeric rating scale (NRS) of neck pain ≥5
5. Voluntary signing of a written informed consent form to participate in the clinical trial

#### 2.2.2 Exclusion criteria

1. Diagnosis of a severe disease that could cause acute neck pain (e.g., spinal metastasis of tumor, acute fracture, spinal dislocation)
2. Progressive neurological defect or severe neurological symptoms
3. Pain caused by soft tissue diseases and not the spine (e.g., tumor, fibromyalgia, rheumatoid arthritis, gout)
4. Other chronic diseases that may hinder the interpretation of the effects or outcomes of treatment (e.g., stroke, myocardial infarction, kidney diseases, diabetic neuropathy, dementia, epilepsy)
5. Current use of steroids, immunesuppressants, antipsychotic drugs, or other drugs that may affect the study outcomes
6. Pregnancy, plan to conceive, or breastfeeding
7. Cervical surgery or procedure in the past 3 weeks
8. Severe mental disorder
9. Less than 1 month since participation in another clinical trial, plan to participate in a study within 12 weeks of enrolment, or plan to participate in another clinical trial during the follow-up period
10. Failure to sign the consent form to participate in the study
11. Other reasons that hinder participation in the clinical trial as determined by the investigators

### 2.3 Interventions

The participants will be randomized into the experimental group and control group. The control group will receive acupuncture, Chuna therapy, pharmacopuncture therapy, and herbal medicine treatments during their hospital stay, while the experimental group will receive NRT in addition to IKMT. All interventions will be performed by Korean medicine doctors with at least 1 year of clinical experience who have been trained for the trial and are familiar with the study procedure.

#### 2.3.1 Experimental group: IKMT + NRT

For the experimental group, four sessions of NRT will be administered from hospital day 2 to 5, and IKMT will be administered daily. Each session of NRT will last for about 10 minutes.

During the NRT, the patient will be seated, and the therapist will apply pressure to the cervical treatment point while firmly supporting the patient's head and progressively extending the neck to move the joint. The therapist will apply pressure to the posterior neck with the thumb and other fingers while supporting the occipital region with the dorsum of the hand.

With the patient seated on a chair, the therapist will place the thumb and index finger on the areas around the articular pillar in the upper cervical region with tense muscles or on the acupoints GB20, BL10, GB21, or SI15; support the back of the patient's head with the 1^st^ and 2^nd^ metacarpal bone; and place the other hand on the patient's forehead. Then, light flexion and extension will be repeated within the ROM of the cervical spine without pain. The weight of the patient's head will add weight to the hand supporting the head and, thus, will transfer pressure to the thumb and index finger, and compression and relaxation will be repeated based on the transferred pressure. During the treatment, the therapist will comfort the patient by assuring them that the treatment does not cause pain so that the patient can relax the neck muscles. Once the targeted area is adequately flexed and extended over a sufficient period of time, the pressure points will be moved to the central and lower neck to repeat the procedure. The range of cervical extension increases at the lower neck area (Figure S1, Supplemental Digital Content).

#### 2.3.2 Control group: IKMT

This group will receive acupuncture, Chuna therapy, pharmacopuncture therapy, and herbal medicine treatments during their hospital stay.

#### 2.3.3 Concomitant treatments

The participants will not be prevented from seeking additional treatment for pain during the study period. They will be instructed to notify the investigator immediately they develop an adverse event or receive additional treatment, which will subsequently be recorded in detail.

### 2.4 Criteria for discontinuing

1. In the case of participant who have been found to have a disease that could affect the decision of the study result that was not discovered at baseline assessment.
2. During the clinical study period, when the participant or participant's legal representative requests to discontinue the study, or the participant withdraws consent.
3. Confirmation during treatment.
4. Existence of a problem in performing medical or oriental medical treatment for neck pain.
5. In case the continuation of participation is not appropriate due to the judgment of the researcher.

### 2.5 Outcome measures

#### 2.5.1 Primary outcome

##### 2.5.1.1 NRS for neck pain

It is a measure of the patient's subjective neck pain. Patients are instructed to choose a number from 0 to 10 that best represents the degree of their current pain (0 for no pain, 10 for the worst imaginable pain).^[15]^

#### 2.5.2 Secondary outcomes

##### 2.5.2.1 NRS for radiating arm pain

It is a measure of patients’ subjective radiating arm pain. Patients are instructed to choose a number from 0 to 10 that best represents the level of their current discomfort (0 for no pain, 10 for the worst imaginable discomfort).^[15,16]^

##### 2.5.2.2 Visual Analogue Scale for (VAS) neck pain and radiating arm pain

The VAS is a 100-mm line with one end indicating no pain and the other end indicating the worst imaginable pain, and patients are instructed to choose a point on the line that indicates their level of pain, which represents their neck pain and radiating arm pain in the past week.^[15,17]^

##### 2.5.2.3 Cervical spine active ROM

The maximum active ROM for the cervical spine (flexion, extension, lateral flexion to the left and right, rotation to the left and right) without pain are measured.

##### 2.5.2.4 Neck Disability Index (NDI)

Functional status will be assessed using the NDI. The NDI was developed as an index for the severity of neck disability. The NDI comprises 10 items, and each item is scored on a scale from 0 to 5. The total possible score is 50. The total score is divided by the number of answered items to compute the average score.^[17,18]^

##### 2.5.2.5 Patient Global Impression of Change (PGIC)

The PGIC allows patients to subjectively assess the level of their improvement on a seven-point scale as follows: 1, very much improved; 2, much improved; 3, minimally improved; 4, no change; 5, minimally worse; 6, much worse; or 7, very much worse.^[19]^

##### 2.5.2.6 Short Form-12 Health Survey version 2 (SF-12 v2)

The SF-12 is a questionnaire for health-related quality of life (HRQoL), and it comprises 12 items in 8 domains (physical functioning, role- physical, bodily pain, general health, vitality, social functioning, role-emotional, and mental health). The questionnaire is completed in about 5 minutes, and a higher score indicates a better HRQoL. The SF-12 is used to assess functional health and well-being of patients and healthy individuals. The Korean version of the SF-12 was validated by Kim et al based on 1000 Korean people for assessing HRQoL.^[20]^

##### 2.5.2.7 The Posttraumatic Stress Disorder Checklist for DSM-5 (PCL-5-K)

The PCL is one of the most widely used self-reported scales for post-traumatic stress disorder. For patients involved in a TA, it is used to assess the level of post-traumatic stress disorder caused by trauma from TAs. We will use the Korean version of the PCL that has been adapted and validated by Kim et al.^[21]^

#### 2.5.3 Drug consumption

The types and doses of drugs taken during the study period (drugs prescribed for current illness or rescue drugs) will be examined based on the questionnaires. The patients will also be instructed to record the frequency of other treatments, such as physiotherapy and injections.

#### 2.5.4 Adverse events (AEs)

AEs refer to undesirable and unintended signs, symptoms, or diseases that occur post-procedurally during the clinical trial (e.g., laboratory abnormalities), and they do not have to be causatively associated with the procedure. The investigators will assess the causality between the treatment modality and each AE based on the 6-level World Health Organization-Uppsala monitoring center causality assessment system (1 = definitely related, 2 = probably related, 3 = possibly related, 4 = probably not related, 5 = definitely not related, and 6 = unknown). In addition, all AEs will be categorized into three severity levels based on the Spilker classification system (Mild (1): does not significantly impair daily activities (function) nor require additional medical intervention; Moderate (2): significantly impairs daily activities (function) and may require additional medical intervention but will resolve; Severe (3): severe AE that requires intense medical intervention and leaves sequela).^[22]^

### 2.6 Participant timeline

At the screening visit, the participants will be enrolled after signing a written consent form, and those who qualify per the inclusion/exclusion criteria will proceed with the trial. The detailed timeline is shown in Table 1.

**Table 1:** Schedule of study.

Time point	Study period						
	Screening	Active treatment				Follow-up	
	Day 1 (visit 1)	Day 2 (visit 2)	Day 3 (visit 3)	Day 4 (visit 4)	Day 5 (visit 5)	Discharge (visit 6)	12 wks (visit 7)
Enrolment							
Eligibility screening	○						
Written informed consent	○						
Sociodemographic hic characteristics	○						
Information about accident	○						
Randomized allocation		○					
Credibility and expectancy questionnaire		○					
Intervention							
IKMT + NRT	Δ (only IKMT)	○	○	○	○	Δ (only IKMT)	
IKMT	○	○	○	○	○	○	
Assessment							
Check symptoms and Med change	○	○	○	○	○	○	○
NRS for neck pain	○	○∗	○∗	○∗	○∗	○	○
VAS for neck pain		○∗	○∗	○∗	○∗	○	
Neck ROM		○∗	○∗	○∗	○∗	○	
NRS for arm pain		○∗	○∗	○∗	○∗	○	○
VAS for arm pain		○∗	○∗	○∗	○∗	○	
NDI		○		○†		○	○
SF-12		○		○†		○	○
PGIC				○†		○	○
PCL-5-K		○		○†		○	○
Adverse events		○	○	○	○	○	○

If Day 5 (visit 5) and day of discharge are the same, then the assessment for Day 5 (visit 5) could be replaced by discharge assessment. The follow-up visit at 12 wks after registration will have a time window of ± 14 days. F/U = follow-up, IKMT = integrative Korean medicine therapy, NDI = neck disability index, NRS = numeric rating scale, NRT = non-resistance technique, PCL-5-K = post-traumatic stress disorder checklist for DSM-5, PGIC = patient global impression of change, ROM = range of motion, SF-12 = 12-item short-form health survey, VAS = visual analogue scale.

∗ Additional evaluation after treatment.

† Investigate only if Day 4 ad 5 (visit 4, 5) and day of discharge are the same.

### 2.7 Sample size

The null hypothesis of this study is that there will be no differences in the improvement of acute neck pain between the experimental group and control group after the additional use of NRT by the experimental group, and the null hypothesis will be tested using analysis of covariance as the primary analysis. The significance level (*α*) was set at 0.05 (two-tailed), type 2 error (*β*) at 0.2, and power at 90% to calculate the sample size. The sample size was determined with reference to a previous pilot study. The Cohens’ *d* was 0.63, and the correlation between the baseline and endpoint measurements was assumed to be 0.5. A total of 41 participants were needed per group. Based on a 30% potential withdrawal rate, the target number of participants was set to 60 for each group.

### 2.8 Allocation and concealment

The same number of patients will be assigned to each groups using a randomization table generated by a statistician that is not directly involved in the study. Block randomization will be used, and the size of each block will be randomly set to 2, 4, or 6. The assignment results will be sealed in an envelope and stored in a double-locked safe until immediately before the initiation of the treatment for the participants who sign the consent form. After opening the envelope, no one can alter the assignment results, and the random number displayed inside the envelope will be assigned to the patient and recorded in the electronic medical records. Only investigators registered for the trial can screen, enroll, and assign a random envelope to the participants.

### 2.9 Blinding (masking)

Due to the nature of the intervention, it will be impossible to blind the participants and the physician, so we will blind the assessor, who is not involved in the screening or intervention, during the outcome measurements.

### 2.10 Data collection, management, and analysis

The standard operating procedure (SOP) will be provided to the investigators, including the assessor and physicians, and they will be trained for the assessments and data entry based on the SOP. Only the investigator who is responsible for data management can access the data recorded on the case report form. The investigators will be in touch with the patients for 12 weeks for the follow-up assessments, and the patients will be allowed to ask study-related questions at any time during the study period by contacting the investigator through the investigator's contact information provided prior to the beginning of the study. Patients who have to discontinue the study will be followed up upon providing consent for follow-up.

### 2.11 Statistical methods

In this study, both the intention-to-treat and per-protocol analyses will be performed, with intention-to-treat as the primary analysis. In the per-protocol analysis, participants who receive at least three sessions of treatment will be analyzed separately. The NRT + IKMT group and IKMT group will be compared, and the aim is to evaluate the superiority of NRT + IKMT.

Missing values will be processed through a mixed model for repeated measures in the primary analysis, and multiple imputations and the last observation carried forward will be performed for sensitivity analysis. For the Kaplan-Meier curve and log-rank test, the patients who drop out will be right censored, and regarding intermittent censoring, we will assume that no event occurred during the specified time period.

The sociodemographic characteristics of the participants and treatment expectancy will be evaluated by group. Continuous variables will be presented as mean (standard deviation) or median (quartile), and the two groups will be compared using the Student *t* test or Wilcoxon-rank sum test depending on the distribution pattern. Categorical variables will be presented as frequency (%) and will be analyzed using chi-square test or Fisher's exact test.

The endpoints of this clinical trial are the changes in the continuous outcomes (NRS, VAS, NDI, SF-12, ROM, PCL-5-K) at each time point from the baseline. A linear mixed model will be used as the primary analysis, and a random intercept model, which includes subject the random effects. Fixed effects will include baseline outcomes and clinically important covariant factors that are statistically significantly differ between groups at baseline as the covariates. In addition, time, group allocation, and the time and group interaction will be included in the model to examine the differences in the amount of change over time between the groups. For sensitivity analysis, we will perform analysis of covariance on the multiple imputations and last observation carried forward sets with the same covariates as those in the primary analysis and the group set as the fixed factor.

To compare the number of changes for each outcome within the study period (during treatment and the entire study period) in both groups, we will calculate the areas under the curve at each time point following randomization. The time from randomization to the point at which NRS reduces by more than half of the score will be compared using the Kaplan-Meier survival analysis, and the curves will be compared using a log-rank test. Additionally, we will use the Cox Proportional Hazard Model to compare hazard ratios. We will perform subgroup analyses and compare the degree of pain improvement between the NRT + IKMT group and the IKMT group.

The significance level will be set at .05 for all analyses. All statistical analyses will be performed using the SAS 9.4 (© SAS Institute, Inc., Cary, NC) or R version 4.1.1 (© The R Foundation for Statistical Computing) software, with the statistical significance set at *P* < .05.

### 2.12 Data monitoring

We will monitor the data to ensure participants’ safety and the integrity of the study data. During monitoring, case records and evidence documents will be compared, and participants’ safety data will be reviewed. The initial monitoring will be performed during participant screening and enrolment, and at least two rounds of monitoring will be performed during the study period. The number of monitoring rounds can be adjusted upon discussion among the investigators. Interim analysis will not be performed during this clinical trial.

### 2.13 Ethics and dissemination

The study protocol, case report form, and informed consent form will be submitted to the Institutional Review Board, and the study will be initiated once it is approved. An amendment of the documents will be approved by the Institutional Review Board in advance, and the documents will be continuously updated. All participating investigators will undergo clinical trials and relevant training and will familiarize themselves with the protocol and SOP. Prior to beginning the trial, the investigators will provide the participants with an information sheet outlining the purpose of the study, timeline, and information to be collected to obtain their informed consent, and a copy of the information sheet will be provided to the participants.

### 2.14 Informed consent

Before the start of the study, the researcher will provide the patients with appropriate information about the study for a sufficient time and fills out the study consent form. The signed consent form of the researcher and the participant is kept by the researcher and a copy is provided to each participant.

### 2.15 Confidentiality

All personal data of the participants will be managed rigorously per the Personal Information Protection Act, and all data collected from the consenting participants will be processed anonymously. If the study data are provided to any third organization for professional data analysis, the data will be de-identified and given arbitrary identification codes. All information regarding this clinical trial, including participants’ personal information, will be retained for 3 years following the completion of the study, after which they will be discarded in accordance with the established protocol.

### 2.16 Ancillary and posttrial care

Participants can contact the sub-investigator at any time regarding a study-related injury or for other medical purposes using the contact information provided in advance. If a participant develops an injury directly due to the study procedure, we will provide the best medical interventions and compensation per the designated trial-related insurance.

## 3 Discussion

NRT can enhance impaired functions and alleviate pain in patients with acute neck pain caused by a TA by helping them overcome the fear of pain and movement and retraining them using muscle stabilization and coordination.^[23,24]^ Technically, NRT is a technique that combines joint mobilization and pressure release techniques. Mobilization is effective for the whiplash syndrome, which is characterized by impaired cervical flexor and extensor functions due to neck injury and pain caused by acceleration and deceleration of a vehicle.^[25]^ Mealy et al reported that patients with a whiplash injury who underwent repetitive and passive movements within tolerable ranges had better outcomes than had their counterparts who simply rested or had cervical braces for the first 2 weeks. These authors utilized small movements within limited amplitudes, with smaller amplitudes for spasms and larger amplitudes for stiffness.^[12]^ A pressure release technique is a common technique used in manual therapy, and Pecos-Martin et al improved the pressure pain threshold and strength of the levator scapulae by applying 60 to 90 seconds of pressure on nuchal muscles.^[8]^

One limitation of this study is that the participants and physicians administering the intervention cannot be blinded due to the nature of the intervention. To address this issue, the assessor will be blinded. This RCT aims to evaluate the effectiveness of NRT in a real-world clinical setting. Considering that the target patient population is patients hospitalized after a TA and that these patients are generally treated through IKMT comprising acupuncture, Chuna therapy, pharmacopuncture, and herbal medicine, we used an add-on design for the interventions, where the control group will receive an IKMT while the experimental group will receive NRT in addition to IKMT. Further, to enroll patients with acute neck pain, we included TA within the past week as one of the inclusion criteria. Helping patients act normally by controlling their pain and fear would facilitate early recovery from residual pain.^[4]^

This study will shed light on the effectiveness and safety of early NRT on acute neck pain caused by a TA. Preventing the chronic progression of pain by effectively reducing pain in early stages will ensure the quick return of patients to their normal lives.^[26]^ Consequently, NRT will be one of the viable treatment options for patients involved in a TA, which will present practical benefits for healthcare-related policy makers and clinicians.

## Author contributions

**Conceptualization:** Suna Kim, Kyoung Sun Park, Jinho Lee, In-Hyuk Ha.

**Data curation:** Da-Hyun Kyeong.

**Investigation:** Suna Kim, Da-Hyun Kyeong, Min-Kyung Kim, Chang-Yeon Kim.

**Resources:** Min-Kyung Kim.

**Supervision:** Kyoung Sun Park, Yun Jae Lee, Jinho Lee, In-Hyuk Ha.

**Writing – original draft:** Suna Kim.

**Writing – review & editing:** Kyoung Sun Park, Yun Jae Lee.

## Supplementary Material

Supplemental Digital Content
