# Supplementary figures and images for: The efficacy and safety of non-resistance manual therapy in inpatients with acute neck pain caused by traffic accidents: A randomized controlled trial
Source: Medicine (Baltimore). 2022 Jun 3;101(22):e29151. doi: 10.1097/MD.0000000000029151 (PMC9276176; doi:10.1097/MD.0000000000029151)

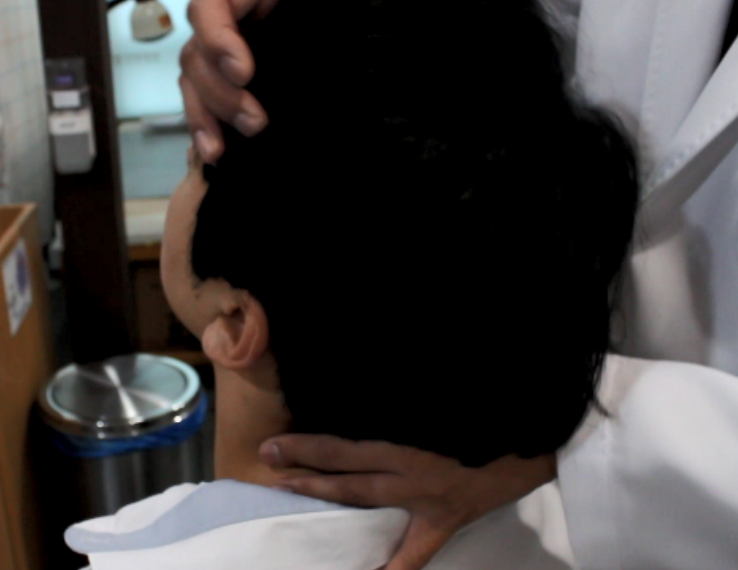


**Supplementary figure 1. Procedure of non-resistance manual therapy**

Supplement: Supplemental Digital Content [file medi-101-e29151-s001.docx]
